# Heterochromatin Morphodynamics in Late Oogenesis and Early Embryogenesis of Mammals

**DOI:** 10.3390/cells9061497

**Published:** 2020-06-19

**Authors:** Irina Bogolyubova, Dmitry Bogolyubov

**Affiliations:** Laboratory of Cell Morphology, Institute of Cytology of the Russian Academy of Sciences, 4 Tikhoretsky ave., 194064 St. Petersburg, Russia; dbogol@mail.ru

**Keywords:** mammalian early development, heterochromatin configuration, karyosphere, oocytes, pre-implantation embryos

## Abstract

During the period of oocyte growth, chromatin undergoes global rearrangements at both morphological and molecular levels. An intriguing feature of oogenesis in some mammalian species is the formation of a heterochromatin ring-shaped structure, called the karyosphere or surrounded “nucleolus”, which is associated with the periphery of the nucleolus-like bodies (NLBs). Morphologically similar heterochromatin structures also form around the nucleolus-precursor bodies (NPBs) in zygotes and persist for several first cleavage divisions in blastomeres. Despite recent progress in our understanding the regulation of gene silencing/expression during early mammalian development, as well as the molecular mechanisms that underlie chromatin condensation and heterochromatin structure, the biological significance of the karyosphere and its counterparts in early embryos is still elusive. We pay attention to both the changes of heterochromatin morphology and to the molecular mechanisms that can affect the configuration and functional activity of chromatin. We briefly discuss how DNA methylation, post-translational histone modifications, alternative histone variants, and some chromatin-associated non-histone proteins may be involved in the formation of peculiar heterochromatin structures intimately associated with NLBs and NPBs, the unique nuclear bodies of oocytes and early embryos.

## 1. Introduction

The development of germ cells, the formation of a zygote and the subsequent cleavage of the embryo are amazing phenomena in nature. The period of oocyte growth at the diplotene stage of the meiotic prophase, the integration of parental genomes after fertilization, which gives rise to a new organism, and the zygotic genome activation (ZGA) at the species-specific stage of early embryonic development are all accompanied by dramatic structural and functional rearrangements of chromatin, both at the morphological and molecular levels.

The morphodynamics and specificity of chromatin rearrangements observed in developing oocytes of several laboratory and farm animals are described in detail (reviewed in [1,2,3,4]). Similar data on chromatin rearrangements in mammalian zygotes and early embryos are less numerous and poorly systematized. Nevertheless, it is clear that the patterns of chromatin distribution in the pronuclei of zygotes and in the nucleus of embryos at the initial stages of cleavage are significantly different from those in the nucleus of differentiated somatic cells.

Rapid development of modern methods especially using improved techniques of sequencing and chromatin mapping [5,6,7,8,9,10] has expanded our knowledge about the mechanisms of chromatin rearrangements during mammalian development and shed light on the features of gene expression and the specific dynamics of the epigenetic landscape of oocytes and early embryos.

The aim of our review is a brief analysis of modern data that can be interpreted in the context of the peculiar chromatin morphology, which had being described by classical morphologists in oocytes and embryos for many decades. We discuss the morphological and molecular features of chromatin changes during mammalian oogenesis and embryogenesis, focusing on the characteristic ring structures of heterochromatin, intimately associated with unique nuclear bodies known as the nucleolus-like bodies (NLBs, in oocytes) or the nucleolus precursor bodies (NPBs, in embryos) that are observed in several species, including mouse and human. Both NLBs and NPBs are sometimes referred to as atypical nucleoli (NCLs) [11].

Oogenesis and embryogenesis are a continuous process, during which the transformations of oocyte chromatin predetermine the outcome of further development after fertilization. Here, we tried to group data on the dynamics of chromatin configuration and its regulatory mechanisms according to the main stages of development, but not according to molecular processes as is typical for reviews on this topic. We found this approach more appropriate to describe the molecular mechanisms that can determine the morphodynamics of heterochromatin, the distribution pattern of which is specific for a certain stage of pro-embryonic and early embryonic development.

## 2. Chromatin Morphology

### 2.1. Oocytes

The specific morphological features of chromatin configurations in growing and fully-grown oocytes had been discussed elsewhere for several mammalian species including mouse, rat, human, monkey, pig, horse, cattle, buffalo, goat, sheep, rabbit, dog, cat, and ferret [2,4,12]. In some mammals including mouse, pig, and human, the gradual condensation and redistribution of chromatin within the oocyte nucleus (germinal vesicle, GV) coincides with the transformation of the nucleolus into a transcriptionally inert nuclear body called the nucleolus-like body (NLB) [13]. In this case, the condensed chromatin forms a more or less compact heterochromatin structure surrounding the NLB in the form of a “ring” or “rim” at the GV stage [14]. The resulting morphologically complex formation is sometimes referred to as a karyosphere [15,16]. It should be noted that the karyosphere is an evolutionarily conserved meiosis-specific structure that represents a “knot” of condensed chromosomes joined together in a limited volume of the oocyte nucleus [16]. It forms at the diplotene stage of meiotic prophase in many animals, from hydra to human. The karyosphere may be a highly complicated structure, with some distinct extrachromosomal elements including NLBs in mammals. Terminologically, according to the definition proposed by Blackman—the pioneer discoverer of a karyosphere—the karyosphere is much more highly organized structure than a karyosome, which is composed exclusively of chromatin [17]. These terminological points have been discussed elsewhere [16].

In spite of the significant differences in the nomenclature proposed for different stages of chromatin rearrangements in the GV oocytes of various animals (Table 1, see also [2]), condensation of chromatin and its relocation to the periphery of a nucleolar remnant, such as the NLB, seem a general tendency in mammalian oogenesis. The most pronounced NLB-associated heterochromatin rings are formed in mouse [18,19], pig [20], and human [21,22] GV oocytes. Contrariwise, goat oocytes ready to ovulate do not contain nucleoli, and therefore NLBs do not appear [23]. Chromatin occupies most of the oocyte nucleus and retains a reticular configuration throughout the entire period of follicle development in cats [24]. In sheep, oocyte chromatin is associated with both the NLBs and the nuclear envelope, exhibiting an unusual SNE (surrounding the nuclear envelope) pattern [25]. In some other mammalian species, e.g., the dog [26,27] and ferret [28], nucleoli/NLBs are embedded into a chromatin mass, but these nucleolar derivatives are not as prominent as the NLBs in mice or human.

**Figure 1:**
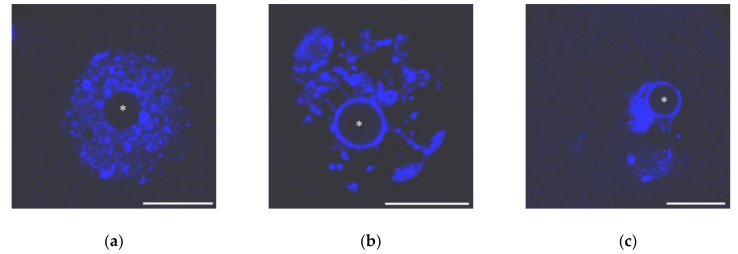
The nuclei of growing mouse oocytes, demonstrating different chromatin organization as viewed after DAPI staining: (**a**) NSN; (**b**) early SN; a heterochromatin “ring” around unstained nucleolus-like body appears; and (**c**) late SN; chromatin is assembled in a more compact mass (karyosphere). Asterisks indicate nucleolus-like bodies. Scale bars represent 20 μm.

Mouse GV oocytes present in antral follicles have different chromatin organization and traditionally are grouped into two main categories: SN (surrounded “nucleolus”) and NSN (non-surrounded “nucleolus”) [30]. In the SN-type oocytes, condensed chromatin completely encompasses the NLB, forming a karyosphere. Conversely, chromatin is less condensed and does not concentrate around the NLB in NSN oocytes (Figure 1). Intermediate pictures of chromatin arrangement, including partially non-surrounded “nucleolus” (pNSN) with several heterochromatin blocks outside the karyosphere, and partially surrounded “nucleolus” (pSN) with incomplete chromatin “ring” around the NLB can be distinguished additionally.

**Figure 2:**
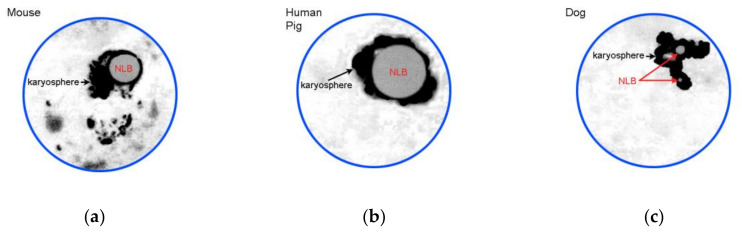
A cartoon illustrating most representative examples of SN chromatin configurations: (**a**) conspicuous nucleolus-like body (NLB) is rimmed by condensed chromatin forming a karyosphere, but some heterochromatin blocks are also located outside; (**b**) all the chromatin is assembled into a rather compact karyosphere around large NLB; and (**c**) configuration similar to (b), but NLBs are not so prominent. Designed according to Figure 1c and data from [20,21,22,27].

The morphology of a heterochromatin mass (karyosphere) in SN or SN-related oocytes is distinct in different mammals. The differences mainly concern the karyosphere compactness and NLB size (Figure 2).

The gradual formation of NSN and SN configurations in mouse oocytes directly correlates with a decrease in the transcriptional activity [18,38]. The intensity of RNA synthesis in the oocyte nucleus depends on the chromatin configuration. In porcine [39,40,41] and bovine oocytes [42,43,44], RNA synthetic activity also gradually decreases while an oocyte grows and almost entirely ceases in SN oocytes. Human SN oocytes are transcriptionally silent, as shown at the light and electron microscopic levels [21,22,45]. It is noteworthy that the RNA synthetic activity of goat oocytes also decreases significantly during oocyte development [46], and the nucleus of goat oocytes is transcriptionally silent before the GV breakdown (GVBD), despite a perinucleolar rim (karyosphere) does not form in this animal [23].

The NSN configuration of chromatin anyhow precedes the SN one. An opposite situation, when SN oocytes might acquire an NSN-like chromatin configuration before the GVBD has not yet been described, with except of a recent study [20] proposed a new classification of the GV chromatin configurations in pig oocytes. In the pig, chromatin undergoes a re-decondensation (RDC) in some of the SN oocytes, forming a RDC configuration.

A striking feature of mammalian oogenesis is that both NSN- and SN-type oocytes can be isolated from the follicles of the same stage, but with a gradual increase in the percentage of SN oocytes in more advanced follicles. For example, the percentage of SN-type oocytes reached 86% in fully-grown antral follicles of rhesus monkeys [34].

However, the SN-type oocytes are observed only in those follicles that have reached a certain stage of folliculogenesis. For example, all mouse GV oocytes from pre-antral follicles exhibit the NSN configuration of chromatin [29,30], while the first SN oocytes usually appear only in the antral follicles of 18-day mice [47]. In oocytes isolated from primary follicles, the SN configuration of chromatin was found only in rabbits, but about half of the oocytes yet had the NSN configuration [32]. In rodents, the SN chromatin configuration (karyosphere) usually appears immediately before ovulation and remains in the GV for 1 h in the rat [48]. In contrast, a karyosphere persists for a significantly longer period, almost all winter, in preovulatory oocytes from antral follicles of the mink [49]. In human oocytes, a remnant of the karyosphere still persists as a single chromatin aggregation for several hours in vitro even following the NLB disassembly and the GVBD, which is clearly observed by time-lapse microscopy [50].

The ratio of NSN- and SN-type oocytes in the same size follicles may depend on the age of the female. For example, GV oocytes of young (two-month old) normally fertile mice might be identified as “canonical” NSN- or SN-type oocytes. In contrast, the oocytes of 11-month-old low-fertility mice had a chromatin configuration that can be referred to as “neither NSN nor SN” (nNSN–nSN) [51]. The nNSN–nSN oocytes exhibited multifarious chromatin configurations: condensed (28%), irregularly distributed in the nucleus (46%), associated with the nucleolus (13%) and clumped (13%).

There are some differences between murine SN and NSN oocytes in terms of the GVBD dynamics and metaphase plate (MI) formation: the GVBD occurs approximately 17 min later in NSN than in SN oocytes, and MI also appears approximately 40 min later [52]. More importantly, SN and NSN oocytes differ in their competence to complete meiosis and develop properly after fertilization. The correlations between the acquisition of the SN chromatin configuration and the ability of oocytes to mature, fertilize successfully, and develop have been documented for different mammals [19,30,34,37,47,53,54]. It is widely assumed that the NSN–SN transition of mouse oocytes is a prerequisite for normal early embryonic development [19,38,55]. In mice, 82% and 45% of SN and NSN oocytes, respectively, can reach metaphase II (MII) [19]. After fertilization, only the descendants of SN oocytes develop properly, while the embryos derived from NSN oocytes are arrested at the two-cell stage [56,57]. The significance of the NSN–SN transition was confirmed by a comparison of the whole transcriptome profiles between mouse NSN and SN oocytes: the mRNA levels of a large set of genes encoding the maternal factors necessary for cleavage and development of the embryo are upregulated in SN oocytes [58].

Ferret oocytes of the SN type were also shown to be more competent for proper development: approximately 75% of ferret SN oocytes can reach MII, while 72.6% of NSN oocytes stop their development at the GV stage before the onset of meiotic divisions [28]. Finally, the SN configuration of chromatin characterized 84.7% of human oocytes developing in vitro [59]. In contrast, human oocytes incapable to resume meiosis after 30 h of in vitro cultivation were characterized by a dispersed or intermediate state of chromatin [60]. The importance of the karyosphere stage for efficient fertilization and further development was confirmed in a study [61] showed that spontaneous Ca^2+^ oscillations and nuclear accumulation of PLC-β1—an important cofactor in the intracellular transduction of many signals—characterize only SN human oocytes with a fully-developed compact karyosphere.

Combelles et al. [33] have distinguished four classes of human oocytes developing in vitro: class A, or pSN (according to the conventional nomenclature for mouse), with additional fibrillar chromatin distributed throughout the nucleus; class B, or SN, with no evidence of chromatin in the remainder of the nucleoplasm; class C, or pSN, with additional chromatin masses in the nucleus; class D, or SN, with additional chromatin threads in the nucleus without evidence of fibrillar patterning like in the class A. In the quoted study, the highest meiotic competence characterized pSN oocytes (class C), which exhibit an incompact karyosphere with some chromatin masses located outside. Conversely, SN oocytes of the classes B and D were abundant in a subpopulation of oocytes that failed to resume meiosis after 48 h in vitro. Thus, the value of chromatin configuration in human oocytes is to be studied further, especially in comparison with animal models, due to the great importance of oocyte quality control for assisted reproduction technologies.

Further evidence that SN oocytes exhibit a higher competence to develop came recently from a study on pig oocytes [20]. As mentioned above, chromatin re-decondensation occurs in porcine SN oocytes immediately before the GVBD, and only these oocytes were able to mature in vitro and support embryo development until the blastocyst stage [20]. In contrast, premature chromatin condensation reduced the developmental potential of oocytes. These observations are consistent with the notion that SN oocytes are most suitable for in vitro fertilization (IVF) [62]. An ultrastructural study on human oocytes [63] confirmed that SN oocytes are of better quality for IVF, as developing in vitro NSN oocytes showed some damage of the cytoplasm.

The formation of the SN chromatin configuration in murine GV oocytes is accompanied by a redistribution of centromeric and pericentromeric heterochromatin [64,65,66,67,68], including the chromocenters—discrete chromatin structures highly enriched in tandem repeats and transposable elements [69]. In oocytes present in primordial and primary follicles and arrested in diplotene, centromeres and chromocenters are preferentially located at the periphery of the nucleus. In growing oocytes, centromeres and chromocenters are initially located in the central part of the nucleus—this corresponds to the NSN chromatin configuration—and then gradually move closer to the NLB [66]. The number of chromocenters decreases from about eight in NSN to four in SN oocytes, and the pericentromeric regions are redistributed to the NLB periphery during the NSN–SN transition, as shown by fluorescence in situ hybridization (FISH) with major satellite (MaSat) DNA probes or immunocytochemistry with antibodies against the heterochromatin protein HP1β [68].

A powerful tool in studies of heterochromatin structure in mammalian oocytes and zygotes is the so-called “enucleolation”—microsurgical removing of NLBs from mouse GV oocytes and NPBs from the pronuclei (PNs) of zygotes followed by NLB/NPB transfer [11]. With the help of this micromanipulation technique it was possible to establish that NLBs/NPBs are indispensable for the regulation of MaSat and minor satellite (MiSat) repeats soon after fertilization and serve as major heterochromatin-organizing structure in oocytes and zygotes in the mouse. Removal of these nuclear organelles resulted in alterations in the expression profile during ZGA due to a profound effect on the regulation of centromeric and pericentromeric DNA sequences [70].

A marker of chromocenters is HP1—the main structural protein of heterochromatin [71]. Two HP1 isoforms—HP1α and HP1β—were localized in the heterochromatin of growing oocytes, with accumulation of HP1β around NLBs during the NSN–SN transition [68,72,73,74]. The data on the presence of HP1α in the NLB-associated heterochromatin ring are somewhat contradictory. The authors of [75] revealed HP1α in these heterochromatin areas, but others [73] have reported that HP1α disappears from pericentromeric chromatin in fully-grown (SN) oocytes. Despite this minor discrepancy, it can be assumed that both HP1 isoforms are involved in heterochromatin transformations in growing oocytes.

It is not surprising that the distribution of chromatin remodeling proteins associated with the pericentromeric heterochromatin changes significantly during the NSN–SN transition. For example, ATRX—α-thalassemia/mental retardation X-linked protein, an ATP-dependent helicase, essential for heterochromatin formation and maintenance during meiosis—is predominantly associated with the NLB-surrounding heterochromatin [76,77]. ATRX is also required for the recruitment of the Death-associated protein 6 (DAXX) to pericentromeric chromatin in preovulatory mouse oocytes [78], indicating a close functional partnership between ATRX and DAXX during chromatin remodeling in oocytes.

Since NLBs are a nucleolar derivative, it is not surprising that rDNA is part of the NLB-associated heterochromatin. The rDNA-containing regions assemble together in NSN oocytes to form several highly condensed foci at the NLB periphery [65,66]. Other heterochromatin areas that do not contain the nucleolus organizer regions (NORs) remain distributed in the nucleoplasm until the SN stage [65]. Interestingly, not only the NLB-associated chromatin, but also the NLBs themselves present in NSN oocytes contain active ribosomal genes. However, the NLBs of SN-type oocytes (i.e., those with a fully assembled karyosphere) contain neither transcribed rDNA nor unprocessed and processed rRNAs [79].

The total number of rDNA-positive zones decreases during NSN–SN transition, but their association to MaSat sequences, in contrast, increases at this time. The vast majority of rDNA signals detecting in SN oocytes are associated with MaSat signals. However, a number of those MaSat signals that are not associated to rDNA, as well as the total number of MaSat signals, also increase during the NSN–SN transition. This allows suggesting that the pericentromeric regions, largely presented by MaSat signals, undergo a decondensation to wrap the NLB, while rDNA sequences become more condensed [68].

Thus, drastic chromatin transformations during mammalian oocyte development involve different functional areas of chromatin, including pericentomeric, centromeric and rDNA-containing regions. As a result, a complex heterochromatin compartment (karyosphere) forms around the NLBs. Numerous data on the large-scale chromatin rearrangements during late mammalian oogenesis allows one to conclude that the NLB-associated heterochromatin formed during the NSN–SN transition is indeed a dynamic hub of many activities. The formation of this unique heterochromatin compartment is necessary for the successful completion of meiosis and subsequent early embryonic development.

### 2.2. Zygote and Pre-Implantation Embryos

The acquisition of a specific morphological configuration by heterochromatin is also characteristic of mouse zygotes, in which the parental genomes are initially separated and exist in two haploid pronuclei (PNs): paternal (pPN) and maternal (mPN). Within the first hours after fertilization, before the formation of the PNs, maternal chromatin looks like a round or elongated mass localized near the polar body. Paternal chromatin retains an oblong shape during this period, resembling the sperm head [80]. The nucleolus precursor bodies (NPBs)—electron-dense rounded structures that are very similar to oocyte NLBs in morphology [81]—appear in both PNs in the early stages of PN formation.

**Figure 3:**
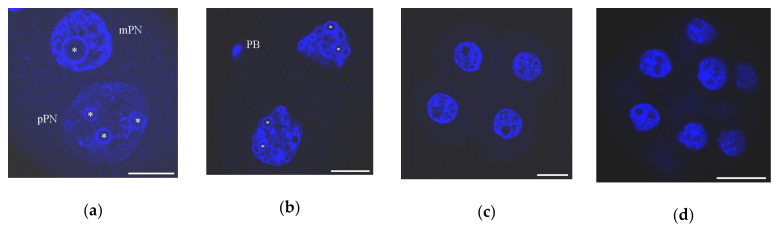
Chromatin organization in mouse early embryos developing in vivo as viewed after DAPI staining: (**a**) zygote, 27 h after peritoneal injection of human chorionic gonadotropin (hCG); mPN, maternal pronucleus; pPN, paternal pronucleus; heterochromatin rings are visible in both PNs around nucleolus-precursor bodies (NPBs); (**b**) two-cell stage, 46 h post-hCG; PB, polar body; chromocenters begin to form at this stage; (**c**) four-cell stage, 55 h post-hCG; numerous chromocenters are visible; heterochromatin rings begin to disappear around some NPBs; and (**d**) morula, 72 h post-hCG. Some typical NPBs are marked by asterisks. Scale bars represent 20 μm.

The main tendency to the formation of a common chromatin landscape in zygotes is the gradual appearance of condensed chromatin regions against the background of more diffuse chromatin, initially dispersed throughout the PN. The NPBs play a role in the formation of the specific regions of heterochromatin and represent the centers around which these regions concentrate. Again, as a result, ring-shaped zones intensely stained with DAPI appear at the NPB periphery in both PNs and persist for several cleavage divisions in the blastomere nucleus (Figure 3). Studies on bovine [82], porcine [83], and human embryos [84] suggest that the spatial arrangement of specific heterochromatin areas at the NPB periphery is a common feature of the beginning stages of cleavage in mammals.

As in fully-grown oocytes where the NLB-associated heterochromatin is enriched with DNA tandem repeats, including centromeric DNA sequences [76], the pericentromeric and centromeric DNA regions are also essential components of the NPB-associated heterochromatin in zygotes and early embryos [85,86,87,88]. In mouse zygotes, pericentromeric chromatin appears around the NPB in the middle of the first cell cycle, termed stage PN2–3 [85,87]. Immediately after fertilization, it quickly organizes around NPBs in the mPN, but remains assembled together in more or less compact masses located centrally in the pPN. At the PN3 stage, only 3% of NPBs in mPNs vs. 30% in pPNs are not associated with the pericentromeres. A more or less complete heterochromatin contour is formed around the NPBs in both pPNs and mPNs at stages PN4–PN5. In addition, a portion of pericentromeric chromatin is localized at the periphery of PNs (in 74% of mPNs and in 96% of pPNs) as extended “filaments” or more compact zones. Some of these pericentromeric filaments extend from the NPB periphery to the periphery of the PN [85].

At the beginning of the second cell cycle, pericentromeric heterochromatin retains in a close association with the periphery of NPBs, forming partial “rims” or, rarely, spherical zones corresponding to the pro-chromocenters. By the end of the second cell cycle, the ratio of these zones changes: the spherical zones become more numerous, and the number of NBPs surrounded by the pericentromeric “rim” decreases. In turn, the regions of centromeric heterochromatin are always associated with both zones of pericentromeric chromatin [85].

At the late two-cell stage of mouse development, DNA tandem repeats begin to regroup into chromocenters [89,90] (reviewed in [91,92]). This process is preceded by a burst of transcription activity of MaSat [90]. Prior to this stage, chromocenters are detected exclusively in polar bodies, but not in the PNs of zygotes, with rare exceptions for some mPNs. Typical chromocenters similar to those in somatic cells are observed at the four-cell stage. They are a compact mass of pericentromeric chromatin with individual centromeres at the periphery. In parallel, there is a decrease in the number of NPBs that transform into functionally active nucleoli and lose their peripheral heterochromatin rims [85].

Studies on animals other than mice have shown that the chronology of the formation of chromocenters is determined by the chronology of ZGA in the particular species. For example, chromocenters appear at the eight-cell stage of bovine development, i.e., before the completion of cattle ZGA [93]. In rabbit, pericentromeric chromatin begins to compact at the four-cell stage, just before ZGA, which occurs between the eight- and sixteen-cell stages [94], and then forms aggregates at the eight- and sixteen-cell stages, i.e., during and after the ZGA [88]. Interestingly, typical heterochromatin rings appear around NPBs in rabbit embryos not earlier than at the four-cell stage, while they are already visible at the one-cell stage of early development of the mouse [80], in which the ZGA completes towards the end of the two-cell stage [95].

The pericentromeric heterochromatin-associated protein ATRX is transmitted from the oocyte to zygote with maternal chromosomes. It is initially colocalized with the perichromatin regions of condensed chromosomes during anaphase II and subsequently localizes to the NPB-associated heterochromatin [76]. Immediately after fertilization, ATRX is detected in heterochromatin structures only in the mPN of mouse zygotes. Later, the level of ATRX gradually decreases and no ATRX staining was detected in both paternal and maternal heterochromatin 10–12 h after fertilization. Beginning from the S phase of the first cell cycle, ATRX associates with the NPB periphery again [70]. This association persists at the two-cell stage, when ATRX is localized both in the NPB-associated chromatin and in the chromocenters. In the morula stage, the ATRX distribution becomes diffusive [96].

ATRX seems to play a role in transcriptional repression of MaSat repeats in the mPN and contributes to the epigenetic asymmetry of pericentromeric heterochromatin in the maternal genome, because of MaSat transcripts were detected in association with the NPB-associated heterochromatin in pPNs, but not mPNs, in the majority of mouse zygotes [97]. As in the oocyte, ATRX is required for the recruitment of DAXX in the pericentromeric heterochromatin, and ATRX-deficient zygotes fail to recruit DAXX to both maternal and paternal heterochromatin [78].

Another essential protein—HP1—maintains the structure of heterochromatin at during early mouse development, but its isoforms (HP1α, HP1β, and HP1γ) appear at different developmental stages: HP1α is not detected until the four-cell stage, HP1β appears already in both PNs at the G1 stage, and HP1γ is detected after the stage S/G2 [98]. The localization pattern of HP1β differs in the pPN and mPN. This HP1 isoform is strongly colocalized with the DAPI-stained heterochromatin in the mPN, but not in the pPN, where HP1β distribution is more diffuse. Lysis experiments showed that a physical association of HP1β with chromatin is stronger in the mPN [99].

Interestingly, HP1β exists in the pPN of mouse zygotes despite the absence of its main binding target—H3K9me3 [100]. Hence, unlike differentiated cells, the zygote is characterized by an atypical interaction between HP1β and H3K9me, which leads to the inability of SUVAR39h—the histone methyl transferase responsible for heterochromatic H3K9 trimethylation—to convert H3K9me into H3K9me3. There is evidence that delayed methylation of H3K9 in the pPN is caused by the inhibitory factors and not by the absence of the enzymatic activity, as suggested earlier [101]. HP1β or other proteins that bind methylated lysine residues pretend to be a role of these factors [100]. However, this hypothesis requires an experimental verification.

Generally, the morphological pictures that reflect the changes of heterochromatin configuration in early embryogenesis resemble those in oogenesis at first glance. The unique nuclear bodies—NPBs and NLBs—serve as a platform around which chromatin condenses to form a peculiar heterochromatin ring containing the areas of centromeres and pericentromeres.

## 3. Molecular Mechanisms of Chromatin Rearrangements

In the oogenesis and early development of mammals, the functional activity of chromatin is largely regulated by the unique epigenetic landscape created by post-replication DNA modifications, post-translational modifications of DNA-associated proteins and ATP-dependent nucleosome remodeling.

One of the main epigenetic mechanisms in the cell is DNA methylation, which changes the binding sites of a number of transcription factors, thereby modulating transcription [102]. Several forms of methylated DNA nucleotides have been discovered. The main modifications are 5-methylcytosine (5mC) and 5-hydroxymethylcytosine (5hmC) generated by oxidation of 5mC. In mammalian somatic cells, DNA methylation is almost exclusively found in CpG dinucleotides, which are often present in CpG-enriched regions of the genome, called the CpG islands [103]. Some methyl-CpG binding proteins maintain the stability of methylated DNA regions. In particular, these proteins recruit histone-modifying complexes to methylated sequences and thereby regulate subsequent chromatin reorganization, stabilize gene expression patterns, and maintain integrity of the genome [104].

Other essential epigenetic marks that play a key role in chromatin remodeling in oocytes and embryos are post-translational modifications of core histones [105,106,107,108]. In particular, the modified histones are involved in the maintenance of an active or repressive state of chromatin and also indirectly affect the chromatin structure by recruiting ATP-dependent chromatin remodeling complexes [109].

In addition, oocytes and early embryos are characterized by a specific pattern of alternative histone variants, providing another layer of chromatin control. Non-canonical histone variants can replace canonical histones, thereby potentially switching the state of chromatin. Replacing the canonical histones with their variants can erase or alter the pattern of histone post-translational modifications [110] to support oogenesis and early development.

### 3.1. Oocytes

#### 3.1.1. DNA Methylation

The epigenetic transitions including DNA methylation in mammalian oogenesis and pre-implantation development are well explored, especially in mouse and human [111]. At the same time, genome wide molecular studies are scarce, including those at single-cell resolution (e.g., [9]), which could help to establish true correlations between specific chromatin morphology (SN and NSN configurations) and the specific DNA methylome in fully-grown oocytes.

The level of global DNA methylation changes significantly during oogenesis. In oocytes from primordial follicles at the beginning of folliculogenesis, DNA is practically unmethylated compared to GV oocytes present in antral follicles, which demonstrate approximately 40% global DNA methylation [112], with a higher level of CpG methylation in SN oocytes [113]. However, experimental conditions preventing DNA methylation in the oocyte do not compromise of fertilization and further development of the embryo until mid-gestation, but DNA methylation is essential for genomic imprinting [111].

At the beginning stages of follicle development, de novo DNA methylation requires a permissive histone modification state. For example, histone H3 must be trimethylated at lysine 36 (H3K36me3), but should not be di- or trimethylated at lysine 4 (H3K4me2/me3) in areas destined for DNA methylation [112,114]. In addition, H3K9me2-enriched chromatin domains usually do not undergo CpG methylation in mouse oocytes, in contrast to embryonic stem and somatic cells [115].

#### 3.1.2. Post-Translational Histone Modifications

If DNA methylation reprogramming is highly similar between mouse and human [116,117], then the reprogramming of histone modifications is much more complex.

Growing mouse oocytes are characterized by numerous post-translational modifications of histones in certain lysine residues. The epigenetic reprogramming of chromatin mostly involves methylated H3 (H3K4me2, H3K4me3, H3K9me2, and H3K9me3) and acetylated H3 and H4 (H3K9ac, H3K18ac, H4K5ac, and H4K12ac) [1,64,113], with their relatively higher levels in SN oocytes. Similar dynamics of histone modifications is observed in the oocytes of other animals, in which the chromatin configuration differs from that in mice by the absence of noticeable heterochromatin rings associated with the NLBs. For example, the specific modifications H4K8ac and H4K12ac are deposited during chromatin condensation in the late equine oocytes [118].

Besides, histone deacetylation plays a role in the regulation of the spatial organization and functional status of oocyte heterochromatin [1]. In mice, the deleting of both (but not separately) *Hdac1* and *Hdac2* genes encoding histone deacetylases results in follicle developmental arrest at the secondary follicle stage [119]. Application of trichostatin A—an inhibitor of histone deacetylases—led to noticeable changes in chromatin configuration in the GV of mouse SN oocytes [64]. Experiments with trichostatin A also confirmed that phosphorylation of histone H3 is another key event during the NSN–SN transition [120].

The predictable reorganization of heterochromatin during oogenesis is impaired under experimental conditions when the specific landscape of histone modifications is disrupted, e.g., after oocyte-specific depletion of mammalian histone methyltransferase G9a (also known as EHMT2), which leads to a decrease in H3K9me2 level and to an impaired SN chromatin structure [115]. On the other hand, impaired H3K4me3 deposition affects the functional activity of heterochromatin, but does not interfere with its structural rearrangements. In particular, no changes in the formation of the SN chromatin configuration were observed with overexpression of the H3K4me3 demethylase KDM5B [6] or with a deficiency of the H3K4 methyltransferase KMT2B/MLL2 [121]. In addition, deletion of the CXXC-type zinc finger protein 1 (CFP1)—the DNA CpG-binding subunit of the SETD1 histone H3K4 methyltransferase complex—caused a decrease in H3K4me3 levels in *Cxxc1* knockout mice, but did not affect the chromatin configuration [122]. However, CFP1 depletion has led to decreased developmental competence of oocytes and female fertility.

The distortion of normal NSN–SN transformation was described in aging oocytes, which coincides with the changes of histone methylation [51]. According to this study, dimethylation of lysines 4, 9, 36, and 79 in histone 3 (H3K4me2, H3K9me2, H3K36me2, and H3K79me2), dimethylation of lysine 20 in histone H4 (H4K20me2), and trimethylation of lysine 9 in histone 3 (H3K9me3) are characteristic of young GV and MII oocytes. At the same time, a significant percentage of old GV and MII oocytes lacked H3K9me3, H3K36me2, H3K79me2, and H4K20me2.

The distribution patterns of five histone modifications (H4K5Ac, H3K4me3, H3K27me3, H3K9me3, and H4K20me3) were studied during the NSN–SN transition of mouse oocytes with the use of high resolution confocal microcopy and 3D-FISH in 3D-preserved nuclei [68]. Significantly, H3K9me3 and H4K20me3, but not H3K4me3 and H4K5ac, were found associated with pericentromeric chromatin and chromocenters, contributing to the NLB-associated heterochromatin structure in SN oocytes.

It has been documented that the NLB-associated heterochromatin “ring” is marked by H3K4me3 and H4K5ac [1,68,74], which are generally associated with transcriptionally permissive chromatin [123]. Besides, the mouse karyosphere demonstrates the presence of H3K27me3 [124]—a marker of repressed heterochromatin [125]. H3K27me3 deposition is mediated by the Polycomb Repressive Complex 2 (PRC2) and inhibited by EZHIP (EZH1/2 Inhibitory Protein)—a gonad-specific cofactor of PRC2, which limits the enzymatic activity of PRC2 but does not interfere with PRC2 recruitment to chromatin [124]. An inactivation of EZHIP in *Ezhip* knockout mice resulted in a global increase in H3K27me2/3 deposition in the late stages of oocyte maturation [124]. The altered H3K27me3-epigenetic content impaired oocyte functionality and female fertility in this case. Since H3K27me3 is involved in Polycomb-mediated gene silencing [126], it is not surprising that this histone modification normally marks the NLB-associated heterochromatin of SN oocytes [68]. The mouse karyosphere also contains H3K9me3 [68,100]—another well-known marker of constitutively repressed heterochromatin [127]. However, H3K9me3 does not colocalize with H3K27me3 there [68].

In human SN-type oocytes, a highly condensed chromatin, which is organized into a compact transcriptionally inert karyosphere closely associated with the NLB [21,22,45], both H3K27me3 and H3K4me3 are markedly deposited in this heterochromatin structure [10,128]. Thus, the histone modifications related to repressed and active chromatin structure can participate in the NSN–SN transition and are deposited in the NLB-associated heterochromatin ring (karyosphere) in SN oocytes.

The karyosphere in mammalian oocytes is a vivid example of a situation when the unpretentious logic “on/off” is too simple to describe the histone code. A class of developmental regulator genes that carry both activating H3K4me3 and repressive H3K27me3 marks have been identified and referred to as “bivalent genes” [129]. They are able to switch chromatin over to an active or silent state.

Several highly sensitive variants of ChIP–seq allowed revealing non-canonical (nc) forms of H3K4me3 and H3K27me3 in mouse oocytes [5,6,7,10]. These ncH3K4me3 and ncH3K27me3 uniquely mark poorly methylated untranscribed regions [130], forming the partially methylated domains (PMDs). It is highly likely that ncH3K4me3 is involved in repression of chromatin transcriptional activity in oocytes (and later in early embryos), in contrast to the canonical form of H3K4me3. Indeed, downregulation of H3K4me3 in mouse fully-grown oocytes by overexpression of the lysine-specific demethylase KDM5B leads to the resumption of the transcriptional activity of heterochromatin in SN oocytes. At the same time, H3K4me3 exhibits a canonical pattern in zebrafish oocytes and hence ncH3K4me3 may be unique to mammals [6]. It is interesting that, unlike that in mouse, the distribution pattern of the permissive mark H3K4me3 largely exhibits canonical patterns at promoters of human oocytes [10].

#### 3.1.3. Essential Non-Histone Proteins Involved in the NSN–SN Transition

Growing oocytes are highly enriched in the poly(rC)-binding protein 1 (PCBP1, also known as hnRNP E1), which plays an important role in controlling gene expression as a transcriptional regulator [131]. Experiments with microinjections of PCBP1-specific siRNAs into mouse oocytes led to disassembly of the karyosphere, restoration of NSN chromatin configuration and resumption of transcription [132]. Thus, PCBP1 is one of the proteins involved in establishing the transcriptionally inactive state of chromatin during karyosphere formation in mammalian SN oocytes. An analysis of the transcriptome of preovulatory oocytes revealed about 4000 transcripts, the number of which increased in the oocytes of *Pcbp1* knockdown mice.

In addition, the developmental pluripotency-associated protein 3 (DPPA3, also known as STELLA) was shown to facilitate transcriptional repression and the subsequent NSN–SN transition in mouse oogenesis [74]. In *Dppa3*-null mouse oocytes, the NSN–SN transition was significantly impaired and transcriptional repression was incomplete.

Chromatin rearrangement during karyosphere formation (the NSN–SN transition) is also regulated by the mitogen-activated proteinkinase (MAPK), as shown in studies of pig oocytes [133]. In this study, a model of complex multifactorial signaling pathways that lead to the formation of the karyosphere was created. In an early stage of oocyte development, a decrease in cAMP activates MAPK, preventing the NSN–SN transition, activating the transcription factor NF-κB, while inhibiting the deacylation of HDAC histones. In the cumulus cells of 1–2-mm follicles, a low level of estradiol and oocyte-derived paracrine factor (ODPF) decreases the level of the natriuretic peptide receptor (NPR2), while increasing the level of the follicle-stimulating hormone (FSH). In turn, FSH increases the level of cAMP, which leads to a decrease in the NPR2 level upon activation of MAPK. Then, MAPK closes the gap junctions, which, along with a decrease in the level of NPR2, decreases cGMP delivery and leads to a decrease in the cAMP level. In large pig follicles, a higher level of estradiol and ODPF, as well as FSH deficiency, initiate a reversal of the above events, which leads to inactivation of MAPK and the formation of the karyosphere.

There is no doubt that the NSN–SN transition is a highly complicated attribute of mammalian oogenesis, which involves many molecular and physiological events. Some of them closely related with the morphological reorganizations of oocyte chromatin are summarized in Table 2.

### 3.2. Zygotes

The pronounced changes in chromatin of maternal and paternal origin occur after fertilization. They are necessary for the integration of parental genomes and for the oocyte-to-embryo transition. Noticeably, there is a clear asymmetry between the pPN and mPN at the zygote stage in many respects.

#### 3.2.1. DNA Demethylation

There are several forms of methylated DNA nucleotides including 5-methylcytosine (5mC) and 5-hydroxymethylcytosine (5hmC)—a modification of 5mC generated by oxidation. The paternal genome is initially characterized by an extremely high degree of methylation. For example, about 80–90% of CpG dinucleotides are methylated in spermatozoa [134,135,136]. The level of methylation of the maternal genome is approximately two times lower [112,137,138]. One of the notable phenomena characterizing the beginning stages of mammalian development is global DNA demethylation required for integration of the parental genomes and further development of the embryo [134,135,139].

Two mechanisms are involved in DNA demethylation during pre-implantation development of mammals: the replication-dependent passive loss of methylation and the active process mediated by methylcytosine dioxygenase TET3 [140]. According to the previous widespread idea, the active demethylation mechanism is intrinsic exclusively for the paternal genome, while the maternal one undergoes the passive DNA demethylation [141,142,143]. However, this concept had been revised and currently there is evidence that the paternal and maternal genomes undergo both passive and active demethylation [116,144].

The level of paternal DNA demethylation after fertilization was previously thought to be significantly higher than that of maternal DNA. This conclusion came from immunofluorescent staining studies, in which anti-5mC signal was not detected in the pPN in the end of the first cell cycle but retained in the mPN [136]. However, the revealing of 5mC by immunocytochemistry was shown to require special conditions including a step of chromatin decondensation. This made it possible to detect 5mC in both mPNs and pPNs throughout the zygotic stage [145,146], indicating the absence of global differences in DNA demethylation between the pPN and mPN.

In mouse zygotes, the NPB-associated regions of heterochromatin contain 5mC and its intermediate modification 5hmC. Moreover, both 5mC and 5hmC are detected simultaneously in both mPNs and pPNs, with no reciprocal change in the levels of these marks [146]. Direct quantification of 5mC and 5hmC levels in mouse pre-implantation embryos by mass spectrometry confirmed that there is no loss of 5mC for 10–48 h after fertilization [147], suggesting the former reciprocal model is invalid. According to the modern notion, 5hmC is not just a simple intermediate in an active demethylation process but could play its own specific role during mammalian early development [148]. A peak of 5hmC is observed in mouse late zygotes, but it is unrelated to any change in 5mC level [148]. The TET3-driven appearance of 5hmC in the mouse pPN was found not linked to the sperm-derived 5mC [149]. Moreover, the accumulation of 5hmC in the zygote is dependent on the activity of DNA methyltransferases DNMT3a and DNMT1, indicating a link between active DNA demethylation and de novo DNA methylation in early mouse embryogenesis [149].

#### 3.2.2. Post-Translational Histone Modifications

The epigenetic asymmetry between the pPN and mPN in mammalian zygotes can also be monitored in respect to the distribution patterns of post-translational histone modifications. In mouse, di- and trimethylated H3K9 modifications are revealed in the mPN, but not the pPN, at stage PN0, despite that monomethylated H3K9me1 and H3K27me1 are revealed in the pPN at this stage [100]. This pattern was completely different from that in somatic cells, where a direct association of H3K9me1 with heterochromatin is not detected. It was found that H3K27me3 begins to deposit in the pPN only after completion of DNA replication (stage PN3–PN4), and this histone modification is associated with the NPB-surrounding heterochromatin [100]. Asymmetry of the PNs in mouse zygotes, established by the absence of di- and trimethylated but not monomethylated histones in the pPN in the early stages of pronuclear formation, was also shown for H3K4 and H4K20 [99,150]. The absence of H3K9me3 and H4K20me3 together with the presence of HP1β and monomethylated H4K20me1 indicates the functional “homogeneity” of paternal chromatin and the absence of canonical euchromatin and heterochromatin in the pPN [99]. Contrariwise, the pPN displays a higher level of H3 and H4 acetylation compared to the mPN [151,152]. For example, H3K64ac is initially revealed exclusively in the pPN (stage PN3), but is present in both PNs beginning from the stage PN4 [152].

There are some species-specific features of the deposition of post-translational histone modifications in mammalian zygotes. A clear asymmetry between the pPN and mPN in relation to the content of H3K27me3 was described in the pig [153,154,155] and cow [156,157]. The H3K9me3 pattern was also asymmetric between the pPN and mPN of horse zygotes [158]. At the same time, the distribution of H3K9me3 in bovine zygotes was rather variable and cannot be considered reliable for determining the parental origin of the PN [157].

Remarkably, the early development is characterized by different dynamics of H3K4me3 and H3K27me3 in human and mouse. A special “priming” form of H3K4me3, which nonetheless are to be distinguished from non-canonical H3K4me3 in mouse, appears in four-cell human embryos, prior to ZGA, in CpG-rich promoters and distal regulatory elements of the genome. H3K27me3 is vice versa depleted after fertilization and is re-established yet afterwards the eight-cell stage. In the mouse, ncH3K4me3 and ncH3K27me3—the non-canonical forms of H3K4me3 and H3K27me3—are present in the GV oocytes and inherited after fertilization. Then, ncH3K4me3 is reprogrammed to the canonical H3K4me3 form upon ZGA, while ncH3K27me3 retains even in blastocysts and resets to the canonical H3K27me3 form in post-implantation embryos only [10].

**Figure 4:**
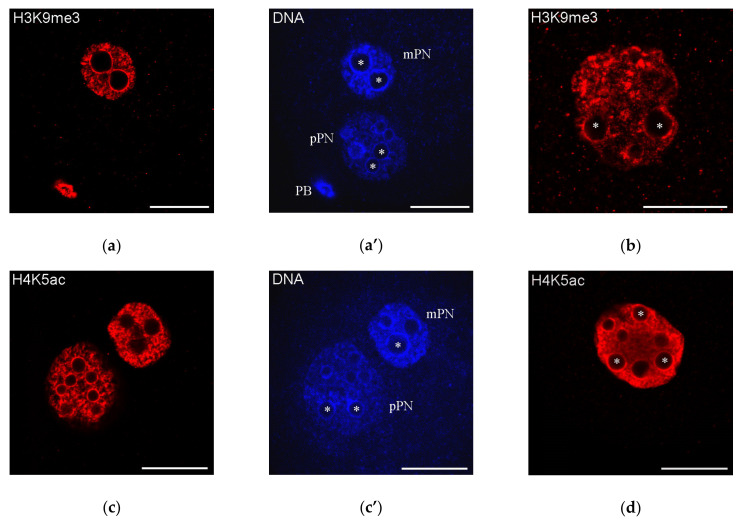
Distribution of H3K9me3 and H4K5ac—the representative marks of “repressed” and “active” chromatin, respectively—in mouse zygotes (**a**,**c**; **a’**,**c’**, **DAPI staining**) and two-cell embryos (**b**,**d**); both marks are detected in the heterochromatin rings around nucleolus precursor bodies (some marked with asterisks); mPN, maternal pronucleus; pPN, paternal pronucleus; PB, polar body. Note that H3K9me3 is revealed in mPN but not in pPN. Scale bars represent 20 μm.

It can be also mentioned that the NPB-associated heterochromatin rings, at least in the mouse, contain the marks of both transcriptionally inert and transcriptionally active chromatin. For example, H3K9me3 and H4K5ac—the representative marks of “repressed” and “active” chromatin, respectively— are detected in these areas [159] (Figure 4), indicating a non-trivial bivalent status of the heterochromatin composing the karyosphere-like structures.

#### 3.2.3. Alternative Histone Variants

##### H3.3

The appearance of the histone variant H3.3 in zygotes is closely related to the replacement of sperm protamines with histones in the pPN. In mice, protamines are removed from sperm chromatin within 30 min after gamete fusion and completely disappear after 50 min [99]. This process occurs before the onset of the S phase of the first cell cycle, and the H3.3 histone variant is used to package paternal DNA into the nucleosomes in a replication-independent manner. The replication-independent mechanism of H3.3 localization can be traced yet in oocytes, in which H3.3 is localized to the NLB-associated heterochromatin, as shown in experiments with microinjections of mRNAs encoding FLAG-tagged H3.3 [122]. In contrast, the canonical histone H3 is included only during DNA replication [91].

It was shown that H3.3 plays a critical role in the regulation of pPN formation in zygotes and significantly affects the development of mouse embryos [99,160,161,162,163,164]. The experiments designed to report H3.3 expression in mice allowed one to establish that sperm-derived H3.3 (sH3.3) is extruded from the paternal genome shortly after fertilization via the second polar body [164]. The maternal H3.3 (mH3.3) abundant in the cytoplasm of mature oocytes [160,161] is incorporated into the paternal genome as early as 2 h after fertilization. Later on, mH3.3 is detectable in the paternal genome until the morula stage [164]. The depletion of mH3.3 in oocytes impaired both the activation of the *Oct4* pluripotency marker gene and global de novo transcription from the paternal genome, which is crucial for early embryonic development.

An important role of H3.3 as a key maternal factor for oocyte reprogramming was confirmed in somatic cell nuclear transfer (SCNT) embryos [165]. It was found that H3.3 is involved in the reprogramming process by remodeling the donor nuclear chromatin through the replacement of donor nucleus-derived H3 with de novo synthesized mH3.3. Knockdown of H3.3 resulted in compromised reprogramming and downregulation of key pluripotency genes, including *Oct4*. Injection of exogenous H3.3 mRNA into oocytes rescued the compromised reprogramming for developmental potentials [165].

In normal mouse development, H3.3 begins to be detected in the pPN earlier than in the mPN [160,161]. This creates an asymmetry of the PNs by the H3.3 content shortly after fertilization. The pPN-specific deposition of H3.3 begins at the PN2 stage, i.e., before the onset of transcription activation, which occurs at stage PN5 [160]. It is noteworthy that H3.3 localizes to the NPB-associated heterochromatin “rings” in the pPN, but not in the mPN, at the start of transcription of pericentromeric repeats [161]. Thus, the distribution of H3.3 once again illustrates the molecular asymmetry of the PNs, the main morphological manifestations of which are presented in Table 3.

It was shown that the histone variant H3.3 and especially H3.3K27 is required to establish the specific heterochromatin structure in early mouse embryos [161]. Indeed, the H3.3K27R mutation resulted in aberrant accumulation of pericentromeric transcripts, mislocalization of HP1β, and developmental arrest. Early mouse development following the zygote stage requires the maternal H3.3 chaperone HIRA [162]. Besides, some other chromatin-associated proteins, such as DAXX, can regulate H3.3 deposition [91]. The accumulation of H3.3 in the pericentromeric regions and the expression of the H3.3-specific chaperone DAXX were reduced in *Dppa3*-null mouse embryos, which are characterized by a violated formation of chromocenters. This indicates that DPPA3/STELLA is a participant of chromatin reorganization in early mouse embryos, apparently by controlling the expression of DAXX [168].

###### H2A Variants

The H2A variants play a key role in the regulation of chromatin activity [169,170]. MacroH2A is a vertebrate-specific histone variant primarily involved in X-chromosome inactivation, but probably may perform other functions. Other two variants—H2A.Z and H2A.X—are highly conserved across various organisms. H2A.Z is involved in transcriptional activation and epigenetic memory, and H2A.X plays a central role in the DNA damage response.

Although the canonical H2A and its variants, including H2A.X, H2A.Z, and macroH2A, are deposited in the nucleus of immature and fully-grown oocytes, as well as on the condensed chromosomes after the GVBD, only H2A.X is abundant in the PNs of mouse zygotes after fertilization, in contrast to the low abundance of canonical H2A and the absence of H2A.Z and macroH2A [171]. The decline in canonical H2A and the removal of H2A.Z and macroH2A histone variants from chromatin after fertilization is required for normal mouse development and may be due to an active mechanism.

Particularly, maternal macroH2A is lost from the zygote at the PN2 stage, reappears in embryos only after the eight-cell stage, and then persists in morulae and blastocysts, where it is revealed in the nucleus of cells both of the trophectoderm and the internal cell mass [72]. These data are in agreement with the notion that macroH2A acts as a barrier to induced pluripotency [172]. Contrariwise, H2A.X—the only abundant H2A variant in the PNs of zygotes—may be involved in maintaining the totipotency of zygotes [171].

####### H1foo

H1foo is an oocyte-specific variant of the linker histone H1, which is expressed in GV oocytes and persists until the late two-cell embryonic stage [173]. H1foo is an epigenomic modulator that decondenses chromatin and impairs pluripotency, because the shRNA-mediated knockdown of *H1foo* recovered the ability of embryonic stem cells to differentiate [174]. H1foo, but not somatic H1, is associated with chromatin in growing, GV-stage, and MII-arrested oocytes. It is also revealed in the PNs of zygotes and in the polar bodies [175]. The experiments with sperm injection into the ooplasm (ICSI) or somatic cell nuclear transfer (SCNT) have shown that H1foo begins to associate with exogenous chromatin of sperm or somatic cell within 5 min and replaces the canonical histone H1 for 60 min. The reverse replacement of H1foo with H1 occurs in the late two- and four-cell stages [175]. H1foo is also involved in the changes of chromatin via nuclear deposition of H3 variants at the one- and two-cell stages of mouse development. Knockdown of the *H1foo* gene led to chromatin condensation and increased deposition of H3.1 and H3.2 at the periphery of zygotic NPBs [176].

## 4. Conclusions

The nucleus of mammalian oocytes and pre-implantation embryos has a unique organization. However, oocytes and zygotes are the cells significantly different in nature: the oocyte is a highly specialized cell, while the zygote is a totipotent cell. Nevertheless, the specific heterochromatin rings form in both late oocytes and zygotes soon after fertilization, contouring the specific nucleolus-related bodies—NLBs and NPBs [159].

Here, we briefly analyzed key epigenetic factors that could determine the morphodynamics of most prominent DAPI-positive structures—the karyosphere and the karyosphere-like rings—in mammalian oocytes, zygotes, and early embryos. In particular, we have discussed how DNA methylation, post-translational histone modifications, alternative histone variants, and some chromatin-associated non-histone proteins may be involved in the formation of these unique heterochromatin structures.

Despite a major advance in our understanding of how differential gene expression is regulated during early mammalian development, a complete picture of the molecular interactions that underlie chromatin reorganization in oogenesis and embryogenesis has not yet been created. Most of the data on the molecular mechanisms of heterochromatin regulation were obtained in studies on a limited number of mammalian species, mainly in the mouse and human. However, both the morphology of heterochromatin and the dynamics of its transformations have species-specific features in oocytes and embryos. Therefore, only the expansion of the spectrum of objects used for molecular analysis can reveal the universal principles of chromatin reorganizations, which are crucial for the onset of the development of a new organism.

Peculiar karyosphere-like structures are indeed a good example of the plasticity and complexity of heterochromatin. To date, some findings force to revise the classical definitions of euchromatin and heterochromatin proposed in the end of 1920s by Heitz (see [177] and references therein) as well as the concept of constitutive and facultative heterochromatin [178], since heterochromatin is not a rigid structure inaccessible to molecules, including transcription factors. The dynamic features of heterochromatin imply a fast diffusion of molecules inside the compartment. It has been shown that heterochromatin proteins, such as HP1, are more mobile than previously thought, and heterochromatin appears to be a surprisingly dynamic compartment [179], even if it forms morphologically stable entities [180]. The dynamic features of the heterochromatin compartment suggest a leading role of phase separation in the formation of heterochromatin [181]. However, it has recently been shown that HP1 demonstrates a weak capacity to form liquid droplets in mouse living fibroblasts [182]. Hence, other factors may apparently be involved in the heterochromatin compaction, toggling between its functional states.

One of the intriguing and practically unexplored problems is the deciphering the mechanisms of interactions between heterochromatin and NLBs/NPBs [183]. The modern concept of membrane-less organelles or biomolecular condensates as liquid droplets [184] allows supposing that the NLBs/NPBs are formed as a result of liquid–liquid phase separation caused by interactions between intrinsically disordered proteins and nucleic acids [185]. In this context, it would be very interesting to establish the role of NLBs and NPBs as a building platform of specific heterochromatin areas, the formation of which is also mediated by phase separation [181].

Further studies in all these and related fields will probably help us to identify the driving forces that determine the morphological singularity of the nucleus during late oogenesis and early embryonic development.

## Figures and Tables

**Table 1:** Main types of non-surrounded “nucleolus” (NSN)-related and surrounded “nucleolus” (SN)-related configurations of chromatin in mammalian germinal vesicle (GV) oocytes, with reference to the original nomenclature including the karyosphere.

Animal	NSN Configurations (No Karyosphere)	SN-Like Configurations		References
		Intermediate Configurations without Prominent NLB-Associated Heterochromatin Rim (Karyosphere), Demonstrating Various Extent of Chromatin Condensation	Chromatin Configurations With Complete Karyosphere	
Mouse	*NSN* Chromatin is not arranged around the NLB and occupies the whole nucleus	*Partly NSN (pNSN)* Some aggregates of chromatin are opposed to the NLB (no karyosphere) *Partly SN (pSN)* A discontinuous heterochromatin rim exists around the NLB (incomplete karyosphere)	*SN* A prominent NLB–heterochromatin complex (karyosphere) exists. Condensed chromatin entirely surrounds the NLB	[14,29,30]
Rat	*Dictyate stage (stage 1)* Chromatin threads are distributed through the nucleus	*Late Dictyate Stage (stage 2)* The “emptiness” of the nucleus is observed (which is apparently due to a karyosphere begins to form)	*Chromatin Mass (stage 3)* Similar to the SN stage (karyosphere) in the mouse	[31]
Rabbit	*NSN* Diffuse, filamentous chromatin is distributed through the nucleus	*SC (singly condensed)* Chromatin is condensed into a single large clump (karyosphere); nucleoli disappeared completely	*Net-Like (NL)* Chromatin is condensed into a net-like structure and surrounds small nucleoli/NLBs (incompact karyosphere) *Loosely Condensed (LC)* Chromatin forms irregularly-shaped clumps scattered throughout the nucleoplasm, or surrounds the nucleoli/NLBs (incompact karyosphere) *Tightly Condensed (NC)* Chromatin further condenses, forming larger clumps with regular edges that are distributed throughout the nucleoplasm or around the nucleoli/NLBs (incompact karyosphere)	[32]
Human	*NSN* Diffusely distributed chromatin	*Class A* The NLB is partially surrounded by chromatin that is also distributed throughout the nucleus (no karyosphere)	*Class B* All the chromatin surrounds the NLB (a fully formed compact karyosphere) *Class C* Chromatin surrounds the NLB; masses of condensed chromatin are also distributed throughout the nucleus (incompact karyosphere) *Class D* The NLB is surrounded by chromatin; threads of dispersed chromatin are distributed throughout the nucleoplasm (incompact karyosphere)	[21,22,33]
Monkey	*GV1* Unrimmed oocytes	*GV2* NLBs are partially rimmed by chromatin (incomplete karyosphere)	*GV3* NLBs are completely rimmed by chromatin (a fully formed compact karyosphere)	[34]
Pig	*NSN* Diffuse and filamentous chromatin is distributed throughout the nuclear area *Prematurely-condensed NSN (cNSN)* Similar to NSN, but chromatin is condensed into solid masses distributed through the nucleoplasm	*Partly NSN (pNSN)* Chromatin begins to condense, particularly in the region around the NLB *Prematurely-Condensed pNSN (cpNSN)* Early NLB-associated heterochromatin rim (karyosphere) is already exists, but many chromatin blocks are also visible outside	*Partly SN (pSN)* Similar to SN, but condensed chromatin is distributed in a wider area of the nucleus (incompact karyosphere) *Prematurely-Condensed pSN (cpSN)* Similar to SN, but single heterochromatin blocks are present outside the karyosphere, resembling mouse SN *SN* All the chromatin surrounds the NLB (a fully formed compact karyosphere), as in human Class B oocytes	[20]
Dog	*Diffuse* Chromatin is homogeneously distributed throughout the nucleoplasm	*Partly Grouped* Chromatin is partly gathered around the nucleolus/NLB (incomplete karyosphere)	*Grouped* Chromatin is restricted to a specific area of the nucleus, surrounding the NLB (a fully formed compact karyosphere)	[27]
Cat ^1^	Chromatin occupies most of the oocyte nucleus, and a reticular chromatin configuration persists during follicular development	N/A		[24]
Cattle	*NSN* Diffuse, filamentous chromatin occupies the whole nuclear volume	*Net-Like (N) Configuration* Condensed chromatin forms a net-like structure in the nucleoplasm, but does not surround the NLBs *Clumped (C) Configuration* Chromatin condensed into large clumps is usually located in the vicinity of the nuclear envelope but does not surround the NLBs *Floccular (F) Configuration* Floccular chromatin is located near the NLBs and nuclear envelope	*SN* NLBs are surrounded by condensed chromatin (karyosphere)	[35]
Sheep	*NSN* Diffuse chromatin occupies the whole nuclear volume	N/A	*SN* Condensed chromatin surrounds the nucleolus *SNE* (specific for sheep) Condensed chromatin is observed near the nucleolus and the nuclear envelope	[25]
Horse	*Fibrillar* Strands of chromatin are located through the nucleoplasm *Intermediate* Strands or irregular chromatin masses occupy over half of nucleus	*Fluorescing Nucleus (FN)* The nucleus displays diffuse or spotty chromatin	*Loosely Condensed Chromatin (LCC)* Looks as an incompact karyosphere *Tightly Condensed Chromatin (TCC)* Chromatin is organized in a single irregular or circular mass, usually surrounding a nucleolar derivative/NLB (compact karyosphere)	[36,37]
Goat ^1^	*GV1* Chromatin is distributed throughout the nucleoplasm, exhibiting a diffuse, filamentous pattern; one or two large nucleoli exist	*GV2 (GV2n/GV2c)* One or two medium sized nucleoli exist; chromatin forms a net-like structure throughout the nucleoplasm (GV2n) or condenses into several large clumps (GV2c) *GV3c* The nucleus contains small nucleoli similar to those of GV3n, but the chromatin is condensed further into several large clumps *GV3n* One or two small nucleoli exist; chromatin condenses into a net-like structure over the nucleoplasm *GV4* (orphan) chromatin is clumped, but no nucleoli are observed	N/A	[23]
Ferret	*FC (fibrillar chromatin)* Chromatin strands occupy most of the nuclear volume	*Intermediate Condensed Chromatin (ICC)* Dense, irregular chromatin masses are distributed throughout the nucleus	*Condensed Chromatin (CC)* Chromatin is highly compact and centered around the nucleolus, forming a compact karyosphere	[28]

^1^ No typical karyosphere found in any stage. N/A, no similar stage determined; NLB, nucleolus-like body; original terms are italicized.

**Table 2:** Key phenomena of the NSN–SN transition

Phenomenon	Main Tendency		Animal	References
Localization of rDNA	Decrease in rDNA-positive zones; increase in their association to MaSat; loss of rDNA transcription machinery from the NLB		mouse	[68,79]
Localization of centromeric and pericentromeric heterochromatin	Moving closer to the NLB; decrease in chromocenter number		mouse	[64,65,66,67,68]
DNA methylation	Increase in CpG methylation level		mouse	[113]
Transcription	Lowering/cessation		mouse	[18,38]
			pig	[39,40,41]
			cattle	[42,43,44]
			human	[21,22,45]
			goat	[23,46]
Histone modifications	Deposition of H3K4me2, H3K4me3, H3K9me2, H3K9me3, H3K9ac, H3K18ac, H4K5ac, and H4K12ac		mouse	[1,64,113]
	Deposition of H4K8ac and H4K12ac		horse	[118]
Localization of some chromatin-associated non-histone proteins	Deposition in NLB-associated heterochromatin	ATRX	mouse	[76,77]
		HP1β		[68,72,73,74]
Meiotic/developmental competence	Improving oocyte quality		mouse	[19,38,55,56,57]
			human	[33,59,60,63]
			ferret	[28]
			pig	[20,62]

**Table 3:** Pronuclear asymmetry in mammalian zygotes

Characteristics		Object	pPN	mPN	References
Presence of histone modifications	H3K9me2	mouse	No	Yes	[100]
	H3K9me3	mouse	No	Yes	[99,100]
		horse	No	Yes	[158]
	H3K27me3	mouse	Yes, after DNA replication	Yes, short time after fertilization	[100]
		pig	No	Yes	[153,154,155]
		cattle	No	Yes	[156,157]
	H4K20me3	mouse	No	Yes	[99]
	H3K64ac	mouse	Yes (PN3)	Yes (PN4)	[152]
Parental level of DNA methylation		mouse	Higher	Lower	[134,135]
		human	Lower	Higher	[166,167]
Presence of alternative histone variants	H3.1/H3.2	mouse	Yes, beginning from the S phase	Yes, before the S phase	[99]
	H3.3	mouse	Yes (PN2)	Yes (PN3)	[99,160,161]
Localization of HP1β		mouse	Diffuse	Predominantly in heterochromatin	[99]
